# Innate Immunity Correlates with Host Fitness in Wild Boar (*Sus scrofa*) Exposed to Classical Swine Fever

**DOI:** 10.1371/journal.pone.0079706

**Published:** 2013-11-18

**Authors:** Sophie Rossi, Anaïs Doucelin, Marie-Frédérique Le Potier, Cyril Eraud, Emmanuelle Gilot-Fromont

**Affiliations:** 1 Office national de la chasse et de la faune sauvage, unité sanitaire de la faune, Gap, France; 2 French Agency for Food Environmental and Occupational Health and Safety, Unité de virologie et immunologie porcine, Ploufragan, France; 3 Université Européenne de Bretagne, Rennes, France; 4 Office national de la chasse et de la faune sauvage, CNERA avifaune migratrice, Chizé, France; 5 Université de Lyon, VetAgro-Sup Campus Vétérinaire, Marcy l'Etoile, France; 6 Université de Lyon, Université Lyon 1, UMR CNRS 5558 – LBBE, Villeurbanne, France; Virginia Tech, United States of America

## Abstract

Constitutive humoral immunity (CHI) is thought to be a first-line of protection against pathogens invading vertebrate hosts. However, clear evidence that CHI correlates with host fitness in natural conditions is still lacking. This study explores the relationship between CHI, measured using a haemagglutination-haemolysis assay (HAHL), and resistance to classical swine fever virus (CSFV) among wild boar piglets. The individual dynamics of HAHL during piglet growth was analysed, using 423 serum samples from 92 piglets repeatedly captured in the absence of CSFV (in 2006) within two areas showing contrasting food availability. Natural antibody levels increased with age, but, in the youngest piglets antibody levels were higher in individuals from areas with the highest food availability. Complement activity depended on natural antibody levels and piglets' body condition. In the presence of CSFV (i.e., in 2005 within one area), serum samples from piglets that were repeatedly captured were used to assess whether piglet HAHL levels affected CSFV status at a later capture. The correlation between CHI and resistance to CSFV was tested using 79 HAHL measures from 23 piglets captured during a CSFV outbreak. Both natural antibodies and complement activity levels measured at a given time correlated negatively to the subsequent probability of becoming viremic. Finally, capture-mark-recapture models showed that piglets with medium/high average complement activity, independently of their age, were significantly less at risk of becoming viremic and more likely to develop a specific immune response than piglets with low complement activity. Additionally, piglets with high average complement activity showed the highest survival prospects. This study provides evidence linking CHI to individual fitness within a natural mammal population. The results also highlight the potential of HAHL assays to explore the dynamics and co-evolution between wildlife mammal hosts and blood-borne parasites interacting with the CHI.

## Introduction

Immunity is an important determinant of vertebrate fitness and host-parasite dynamics [1], [2]. However, the immune system and its relationships with fitness components are not easy to study, partly because of the complexity of immunity and the fact that different pathogens may be differently affected by the many components of the immune system of vertebrate hosts [3]. Although several methods have been proposed to assess immune functions in natural populations, clear demonstrations are lacking that these indicators correlate with disease risk, resistance to infection and host fitness, especially in large and medium sized mammal species [4], [5]. Furthermore, few assays are compatible with the constraints researchers face when capturing wild animals in the natural environment. For example, obtaining repeated samples from the same individual within a short period of time requires repeat captures or maintaining animals in captivity. Moreover, the relevance of these assays has been recently questioned: a single measure of one trait does not reflect the whole individual immunocompetence since the immune function comprises many traits and each individual trait may vary over time at the individual level [3], [6], [7]. Providing evidence that an indicator of immunocompetence correlates with disease resistance and host fitness would emphasise the importance of individual susceptibility as a cause of disease, while validating the use of the considered indicator.

The Constitutive Humoral Immunity (CHI) comprises the non-specific fraction of the humoral (*i.e*., non-cell mediated) immune system, mainly the natural antibodies (Nabs) and the lytic chain complement (Cp), two immune effectors acting in the earliest phase of the innate immunity [8]. This function is considered as largely innate as it develops before any exposure to pathogens and is not targeting a particular pathogen/antigen, as opposed to the specific immunity (*e.g*., antibodies specifically targeting one pathogen) [9]. CHI is considered as an important determinant of host-parasite dynamics providing a first-line of protection against invading pathogens, especially when the infection is disseminated through the blood as for many viruses and bacteria [8]. The link between individual CHI and further resistance to infectious diseases and better survival has been demonstrated through experiments in domestic animals selected for different immune profiles (mainly mutant mice and selected lines of chicken) [10], [11], [12], [13]. Recently a simple method, the haemagglutination-haemolysis assay (HAHL), has been developed to explore the Nabs and Cp levels using the sera from wild birds and mammals [5], [14]. Recent studies provide evidence for the costs and possible trade-offs associated with this component of immunity [15], [16]. However, within natural vertebrate populations, the evidence that CHI variation between individuals influences their resistance to pathogen and fitness is still lacking.

The European Wild Boar (*Sus scrofa* sp.) is a relevant biological model to investigate both the variations and consequences of immunity. This species is indeed abundant in varied ecosystems worldwide and is thus exposed to a large range of pathogens and quality of habitat [17]. Furthermore, host-parasite dynamics have been extensively explored in wild boar since that species shares pathogens with livestock and humans [18], [19]. The classical swine fever virus (CSFV) is among the most virulent pathogens in this species, and is particularly lethal in piglets. After a first phase of multiplication in the tonsils, CSFV invades its host through the blood causing a large range of symptoms including death, which may occur in 80% of infected piglets within naïve populations [20]. Within immunized populations, maternal derived antibodies transmitted before and/or after birth through the placenta and colostrum, may also partially protect piglets until three months after birth [21], [22]. Individuals surviving infection develop their own specific neutralizing antibodies that protect them for life [23]. The varied outcomes of CSFV infection in non-protected piglets suggest that piglets contrast in their receptivity and susceptibility to infection, before developing any specific immunity. Individual susceptibility is influenced by the genetic background and age in the domestic pigs [23], [24]. However, factors influencing individual resistance or recovery and subsequent survival to CSFV infection in nature have been poorly investigated.

The present study aimed at testing the hypothesis that the variation of CHI amongst individuals influences the individual outcome of CSFV infection and subsequent survival. The variation of CHI among and within individuals was first explored in live-trapped wild boar piglets inhabiting two areas with contrasting food availability using HAHL assay on sera collected in the absence of CSFV. The causative relationship between CHI and resistance or recovery to CSFV was then explored using capture histories of piglets during a CSFV outbreak using linear-mixed modelling and capture-mark-recapture modelling [25], [26]. The study was particularly aimed at testing the hypothesis that individuals with the highest CHI levels exhibit the highest resistance or rates of recovery to CSFV infection and had the highest prospects for survival.

## Materials and Methods

### (a) Study areas

The study was carried out at two distinct sites: the Petite Pierre National Reserve (PPNR) (48.5°N, 7.0°E) and the Bitche military camp (BMC) (49.0°N, 7.3°E). These two unfenced 3000 ha areas are located in the Vosges mountains (North-eastern France) [27], [28]. Even though the two sites are located in the same eco-region, these two populations are distant by more than 60 Km and are poorly connected (*e.g.*, no cross captures among more than 500 marked animals for 10 years within the two areas, Rossi unpublished data). The habitat of PPNR is dominated by silver fir (*Abies alba*), Douglas fir (*Pseudotsuga douglasii*), Norway spruce (*Picea abies*) and European beech (*Fagus sylvatica*) [27], whereas BMC is characterized by a more diversified habitat including oak trees (*Quercus rubra*), supplementary feeding (dried corn) and crops (corn). The two populations have similar densities of wild boar (∼10 individuals per km^2^) but previous studies showed that the average growth and survival rates of piglets were lower in PPNR (growth rate = 0.407 Kg/week, 95% confidence interval [0.388; 0.426] and survival rate = 0.901/week, 95% confidence interval [0.880; 0.919]) compared to BMC (growth rate = 0.548 Kg/week, 95% confidence interval [0.527; 0.570] and survival rate = 0.988/week, 95% confidence interval [0.957; 0.997]), possibly due to a lower food availability (Rossi unpublished data). CSFV spread naturally in 2004 within BMC and in 2005 within PPNR, but was not detected again within these two areas after the initial outbreaks [28]. During the 2005 outbreak in PPNR, CSFV dynamics was intensively monitored in recaptured piglets and the average proportion of infected piglets was about 15% [20].

### (b) Captures

Captures were performed weekly according to the process described by Rossi et al. [20] from 18^th^ May to 30^th^ July 2005 in the PPNR and from 9^th^ May to 30^th^ August 2006 in PPNR and BMC. In order to maximize the probability of capturing different individuals, 20 traps (10 traps per area) were set in different valleys using box traps specifically adapted for catching piglets [29].

Each trapped animal was marked with two ear-tags to allow individual identification. Blood samples were taken from the jugular vein in order to explore the dynamics of CSFV virus, antibodies to CSFV and constitutive humoral immunity. Body mass was measured at a precision of 100 grams. Several serum samples and body mass measures from individuals repeatedly captured were used to determine (i) the dynamic of HAHL during piglets' growth according to different environmental conditions and in absence of CSFV (i.e., in 2006 in PPNR and BMC), (ii) whether the HAHL levels affect the CSFV status at a later capture during the 2005 outbreak in PPNR. In order to limit animal handling and stress, a maximum of one blood sample and one weight measurement was performed per animal per week. No anaesthesia was used and all piglets were released alive immediately after handling.

In France, wild boar is a game species neither protected nor endangered. Permission to capture and blood sample the boar was given by the local Direction of Agriculture and Forest of each district (Moselle and Bas-Rhin districts). The authority of the Bitche Military Camp (*i.e*., the French Army) also authorized the study. Since the Petite Pierre National Reserve is managed by the National Wildlife and Hunting Agency (who performed the present study), together with the National Forest Agency, no particular authorisation from the land owner was required. Experimentations were conducted in accordance with European and French legislations on Laboratory Animal Care and Use (French Decree 2001-464 and European Directive CEE86/609). All staff were qualified through mandatory trainings and the sampling was carried out under veterinary supervision.

In order to take into account possible differences in the distribution of births between the two areas, we referred to a previous study which estimated the age of foeti and consecutive birth date in sows hunted from November 2005 to February 2006 (according to the method of Hugget and Widdas [30] and supposing a pregnancy of 115 days) (Rossi, unpublished data). According to this study, the two areas exhibited similar birth distributions in 2006. We thus considered the calendar week of capture to be a good representative for piglets' age in both areas in 2006 and in PPNR in 2005. Piglets' body mass was used as an index of individual body condition at each capture event after correcting for piglets' age (i.e., the calendar week of capture) and area (*i.e.*, different intercept and slopes per area).

### (c) Definition of disease states

The disease status of piglets regarding CSFV was determined by detecting the CSFV genome and specific antibodies. The CSFV genome was first amplified by real-time polymerase chain reaction (r-RT-PCR) using a commercial kit (TAQVET or ADIAVET) according to manufacturer's instructions [31], [32]. To confirm that piglets were viremic at the time of capture (*i.e.*, carrying viral particles in their blood), virus isolation was performed in the PCR positive samples at the French Reference Laboratory for CSF (ANSES) according to the EU-Diagnostic Manual for CSF (Decision 2002/106/EC). The detection of specific antibodies targeting the CSFV was achieved using ELISA kits according to the manufacturer's instructions (Herdcheck CSFV Antibody test kit or CHEKIT CSF SERO Antibody, both distributed by IDEXX and having similar sensitivities). Animals were finally classified into three disease states at each capture event [20]:

- SU: susceptible individuals not protected by specific antibodies (*i.e*., seronegative and vironegative individuals).
- VIR: viremic individuals (*i.e*., virus particles detected in blood either in seropositive or seronegative individual).
- IM: immune individuals protected (at least partially) by specific antibodies targeting CSFV infection (*i.e*., seropositive [specific antibodies in the blood] but vironegative individuals [no virus particles in the blood]). Specific antibodies could be either transmitted by the mother (maternally derived antibodies detected until 3-4 months of age) or actively produced after piglet's infection and recovery.

### (d) Haemagglutination-haemolysis assay (HAHL)

Serum samples (stored at −20°C) were used for measuring the concentration of Nab and Cp activity using a haemagglutination-haemolysis (HAHL) assay. HA level was interpreted as an indicator of Nab concentration and HL was interpreted as an indicator of Cp activity. We used the protocol proposed by Maston et al. [14], modified for mammal species by using chicken red blood cells as target cells, and incubating cells at 37°C [5]. Sera were diluted twofold from 1 to 1/128 in 96-wells round bottom assay plates, and mixed with an equal volume of a solution of 1% chicken red blood cells. After 90 min of incubation, plates were stored for 20 min at room temperature on a 45° sloping surface and scanned for reading haemagglutination (HA) titers. Plates were subsequently stored for 70 min at room temperature on a horizontal surface and again scanned for reading haemolysis (HL) titers. Each plate included a positive standardised sample (diluted serum of rabbit immunized against chicken red blood cells). HAHL titers were determined as the log2 of the last dilution exhibiting HA or HL. Since the antigenic properties of chicken red blood cells vary among chicken lineages [33], the batch of chicken red blood cells could potentially influence the results. We thus randomized the order of the samples to avoid confusion. To exclude potential observer effects, all images were scored by the same trained observer; thus correction for observer effect was not necessary in the subsequent analyses.

### (e) Analysis of HAHL variation using linear mixed modelling

Data from both populations in 2006 were considered for analysing the variations of individual HAHL measures according to piglets' age, body condition and study area, in a context of absence of CSFV. Analyses were performed using mixed linear modelling for taking into account grouping factors such as individual piglets recaptured several times and antigen batches [26]. Piglets' body condition (*i.e*., residuals of the linear model predicting a different growth rate per area), week of capture (proxy of age) and study area (PPNR of BMC) were considered as fixed variables. HA was also systematically included as a potential variable of HL.

Data collected in 2005 in PPNR were used for analysing the relationship between HAHL level at a given capture and the probability of becoming subsequently viremic. A proxy of the probability of becoming viremic between the considered capture and the last capture was estimated by the contrast between the status at a given capture event (1 if piglet was classified as VIR, 0 otherwise) and the final infectious outcome of individual (1 if piglet was classified as VIR at least once during the following captures, 0 otherwise). Linear mixed models considering HA or HL as dependent variables were used for testing the effect of the individual piglet (random variable), the probability of becoming viremic, the week of capture, the body condition and HA measure observed at the same time of HL (fixed variables).

Model selection was based on the Akaike Information Criterion (AIC) computed according to the maximum likelihood method [26]. When the difference in AIC was less than two, the most parsimonious model was selected. We adopted a non-automatic backward procedure starting from a model including all factors and all possible two-way interactions (i.e., between two variables). We first tested random effects and then the fixed effects for taking into account that the intercept (*i.e*., initial levels of HAHL) and slopes (*i.e*., variation over week of capture) were potentially different between blocks of data (*i.e*., individual piglets and antigen batches). Parameters of the best model were finally estimated using the restricted maximum likelihood (REML) method as recommended by Zuur et al. [26]. The significance of the coefficients (for fixed effects) was then tested using Wald tests at the threshold of p = 0.05. Analyses were performed using the lmer function (lme4 package) of the R software version 2.13.2 [34].

### (f) Correlation between Individual HAHL and fitness

**Figure 1:**
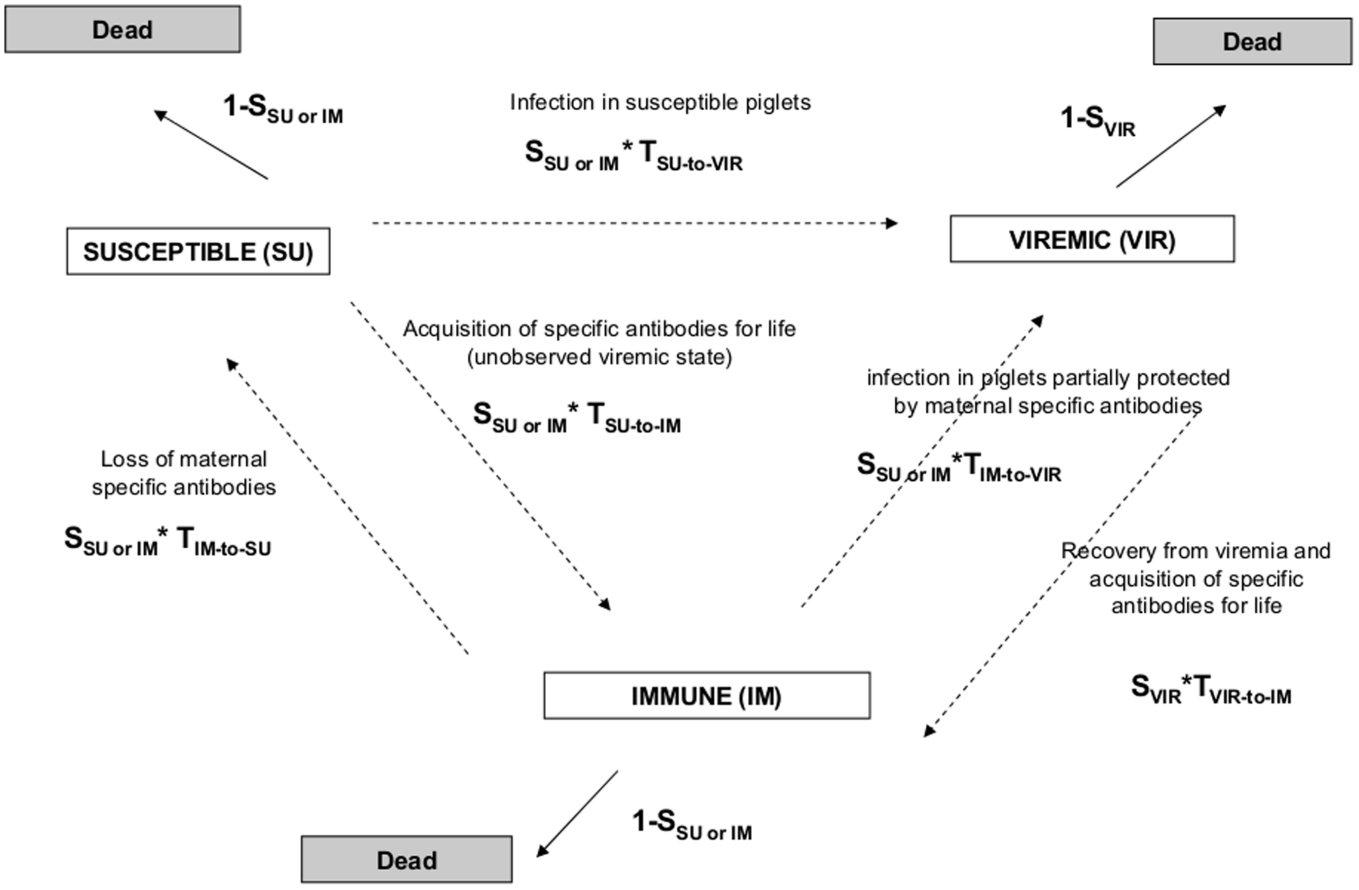
Transitions between the states (SU, IM, VIR, Dead) experienced by piglets from week to week. The transition from viremic (VIR) to susceptible (SU) is not possible since the recovery from viremia is associated with the acquisition of life-long specific antibodies (IM). According to previous results [20], the survival was similar between immune (IM) and susceptible (SU) piglets (SU _SU or IM_) but was lower in viremic piglets (S _VIR_) compared to other categories.

We analysed the effect of individual levels of HA or HL on the probabilities of becoming viremic, to acquire specific antibodies and to survive using a multi-event capture-mark-recapture (CMR) modelling [25]. Multi-event CMR models are multiplicative multinomial models aiming to estimate at each time step the probability of surviving in a given state, the probability of moving from state to state (SU, IM, VIR) and the probability of being captured (Fig. 1). Model parameters were estimated according to the principle of maximum likelihood using an iterative process between the model and the individual histories (see Rossi et al. [20] for more details on the modelling process). The analysis focused on piglets captured in PPNR in 2005, *i.e*., during the period of CSFV outbreak, and for which HAHL measures could be performed. The disease state was included as an explanatory variable for survival since CSFV is known as highly lethal in that age class: survival was expected to be lower in infected piglets compared to susceptible or immune ones (Rossi et al. [20]). We also tested for correlations between HAHL levels and the probabilities of moving between the states (SU, IM, VIR), and for correlations between HAHL and survival. Piglets were considered having “low” (versus “medium-high”) Nabs or Cp concentrations when their average HA or HL levels (after correcting for the effect of the week of capture) was lower than the average HA or HL titers observed at the population level. The goodness of fit of the full multi-event model to data was explored using the program U-Care 2.3.2 (freeware available at http://www.cefe.cnrs.fr/biostatistiques-et-biologie-des-populations/logiciels), according to the process described by Choquet et al. [35]. Then, taking into account the GOF analysis, the multi-event modelling was performed using E-SURGE 1.7 (freeware available at http://www.cefe.cnrs.fr/biostatistiques-et-biologie-des-populations/logiciels) [36]. The model selection was based on the Akaike Information Criterion corrected for small sample size (AICc). When the difference in AICc was less than two, the most parsimonious model was selected (for inference) [37]. Once the model selection was achieved, significant differences between specific parameters of the “best model” were tested using Wald tests at the threshold of p = 0.05 using E-Surge 1.7 such as recommended by Choquet & Nogue [36].

## Results

### (a) Variations of HAHL in growing piglets

**Figure 2:**
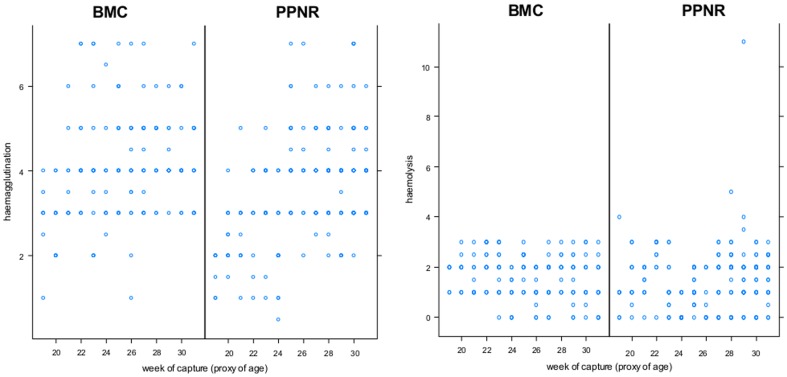
Increase of HAHL levels according to the week of capture (proxy for piglet age) in PPNR and BMC areas in 2006 (in absence of CSFV).

Analyses were based on 423 HAHL measures collected in 92 piglets captured in 2006: 60 individuals from PPNR and 32 individuals from BMC. HA titers ranged from 0.5 to 7 and HL titers ranged from 0 to 5. The retained model for HA (i.e., with the lowest AICc in table 1) included the fixed effects of the week of capture, area where they were captured from, body condition, the interaction between the week of capture and area where they were captured from, and the random effects of the individual piglet and antigen batch. HA titers observed at first capture were significantly lower in PPNR compared to BMC (delta-HA_PPNR/BMC_ = −2.390; 95% confidence interval [−2.092; −0.567]). However, HA titers increased with piglets' age according to a higher slope in PPNR (delta-HA_per-week_ = +0.155; 95% confidence interval [+0.134; +0.175]) compared to BMC (delta-HA_per-week_ = +0.084; 95% confidence interval [+0.062; +0.106]) (Fig. 2). In both areas, after correcting for age, HA titers were positively correlated to individual body condition (delta-HA_per-Kg_ = +0.094; 95% confidence interval [+0.063; +0.124]). The retained model for HL (i.e., with the lowest AICc in table 1) included the random effects of the individual piglet and antigen batch and the fixed effects of HA (delta-HL_per-HA-titer_ = +0.107; 95% confidence interval [+0.070; +0.143]) and body condition (delta-HL_per-Kg_ = +0.049 titers; 95% confidence interval [+0.026; +0.072]) (Table 1).

**Table 1:** AIC of the linear mixed models considered for testing the variation of haemagglutination (HA) and haemolysis (HL) in PPNR and BMC in 2006.

DEPENDENT VARIABLE	INDEPENDENT VARIABLES TESTED	AIC	DELTA-AIC
HA	(week+body condition)*area+*(week+body condition|id_animal)+(1|batch)*	1265	+8
	(week+body condition)*area +*(body condition|id_animal)+ (1|batch)*	1260	+3
	(week+body condition)*area +*(week|id_animal)+(1|batch)*	1260	+3
	(week+body condition)*area +*(1|id_animal)+(1|batch)*	1256	−1
	(week+body condition)*area +*(1|batch)*	1272	+15
	(week+body condition)*area +*(1|id_animal)*	1259	+2
	week + body condition*area +*(1|id_animal)+(1|batch)*	1259	+2
	week*area +body condition +(1|id_animal)+(1|batch)	**1257**	**0**
	week+area +body condition +(1|id_animal)+(1|batch)	1260	+3
HL	HA+week+body condition+area+*(1|id_animal)+(1|batch)*	1084	+1
	HA+week+body condition+area+*(1|batch)*	1087	+4
	HA+week+body condition+area*+(1|id_animal)*	1104	+21
	HA+week+body condition+*(1|id_animal)+(1|batch)*	1084	+1
	HA+week+ area+*(1|id_animal)+(1|batch)*	1087	+4
	week+body condition+area+*(1|id_animal)+(1|batch)*	1090	+7
	HA+ body condition+area+*(1|id_animal)+(1|batch)*	1083	0
	**HA+ body condition+** ***(1|id_animal)+(1|batch)***	**1083**	**0**
	HA+*(1|id_animal)+(1|batch)*	1085	+2
	*Body condition+(1|id_animal)+(1|batch)*	1089	+6

The random effects are indicated in italic and the retained model is indicated in bold.

### (b) Correlation between HAHL and the probability of becoming viremic

Analyses were based on 79 HAHL measures collected in 23 piglets captured in 2005 in PPNR. HA ranged from 1.5 to 7 and HL ranged from 0 to 2.

The probability of becoming viremic was negatively correlated to HA and HL (Table 2). As shown by the comparisons between models HA-2 and HA-4 and between models HL-4 and HL-7, piglets that became viremic between two captures exhibited significantly lower HA and HL titres at the first capture compared to piglets that did not become viremic (delta-HA_CSFV_ = −0.775; 95% confidence interval [−1.459; −0.091]; delta-HL_CSFV_ = −0.442; 95% confidence interval [−0.800; −0.082]). However, this negative correlation was no longer significant when the effect of piglet age was taken into account, since the best models for HA and HL (HA-3 and HL-5 in Table 2) included the effect of week of capture alone.

**Table 2:** AIC of the linear mixed models considered for testing the relationships between haemagglutination (HA) or haemolysis (HL) values (PPNR area in 2005), the probability of becoming viropositive and piglets' age. The random effect of individual piglet is indicated in italic and the best models are indicated in bold.

DEPENDENT VARIABLE	MODEL	INDEPENDENT VARIABLES TESTED	AIC	Delta-AIC
**HA**	HA-1	probability of becoming viropositive + week +*(1|id_animal)*	241.3	+0.1
	HA-2	probability of becoming viropositive+*(1|id_animal)*	247.0	+5.6
	**HA-3**	**week+** ***(1|id_animal)***	**241.4**	**0**
	HA-4	*(1|id_animal)*	249.9	+8.5
**HL**	HL-1	HA + probability of becoming viropositive + week + (*1|id_animal*)	138.7	+0.8
	HL-2	HA + probability of becoming viropositive +*(1|id_animal)*	144.5	+6.6
	HL-3	HA +week +*(1|id_animal)*	138.7	+0.8
	HL-4	probability of becoming viropositive +*(1|id_animal)*	145.4	+7.6
	**HL-5**	**week +** ***(1|id_animal)***	**137.9**	**0**
	HL-6	HA+*(1|id_animal)*	146.3	+8.4
	HL-7	(*1|id_animal*)	149.1	+11.2

### (c) Effect of HL on survival and disease outcome

CMR multi-event models were fitted using 142 capture events from the same 23 piglets measured for HAHL. Among these, 13 individuals exhibited low HA levels (versus 10 with medium-high HA) and 11 individuals exhibited low HL levels (versus 12 with medium-high HL). Goodness-of-fit tests were not significant, thus suggesting a good fit of models to observed data. The retained model included the effect of the disease state on the weekly survival probability and the effect of individual HL (but not HA) on the weekly movement probabilities between states (model 2, Table 3). As expected, viremic individuals exhibited a lower survival rate (S_VIR_ = 0.515; 95% CI [0.232; 0.788]) compared to immune or susceptible ones (S_SU&IM_ = 1.000; 95% CI [0.999; 1.000]). Examining Table 3, it is noticeable that survival was positively influenced by individual HL when no effect of HL on movement probabilities was considered, as the model [SU vs. VIR] + HL (model 3) has a lower AIC value than [SU vs. VIR] (model 4, delta AICc = 2.9). However, when considering the effect of HL on movement probabilities between states, the effect of HL on survival was no longer significant: the delta-AICc between models including or not including HL effect on survival (models 1 and 2) was 1.4 (Table 3). This comparison suggests that HL influenced survival by affecting the probability of becoming viremic or immune rather than through other (unexplored) mechanisms.

**Table 3:** AICc of the multi-event capture-mark-recapture models considered for testing the effect of haemolysis (HL) (two groups: low or medium-high haemolysis) and haemagglutination (HA) on survival and movement between disease states. The retained model is indicated in bold.

MODEL	COVARIABLES FOR SURVIVAL	COVARIABLES FOR MOVEMENT BETWEEN STATES	NUMBER OF PARAMETERS	DEVIANCE	AICc	DELTA-AICc
1	SU versus VIR+HL	HL	16	475.30	512.0	−1.4
**2**	**SU versus VIR**	**HL**	**15**	**479.28**	**513.4**	**0**
3	SU versus VIR+HL	/	11	491.61	515.8	+2.4
4	SU versus VIR	/	10	496.83	518.7	+5.3
5	SU versus VIR+HA	/	11	496.66	520.9	+7.5
6	SU versus VIR	HA	15	491.52	525.7	+12.3
7	SU versus VIR+HA	HA	16	490.98	527.7	−14.3

The probability of becoming viremic and of acquiring antibodies was also influenced by HL. Among the susceptible piglets without specific antibodies (SU), individuals with medium-high HL were less at risk of becoming viremic (T_SU-VIR_ = 0.044; 95% CI [0.011; 0.165]) compared to piglets with low HL (T_SU-VIR_ = 0.297; 95% CI [0.154; 0.495]). Piglets with medium-high HL were also more likely to acquire specific antibodies (T_SU-IM_ = 0.100; 95% CI [0.041; 0.226]) compared to piglets with low HL (T_SU-IM_ ∼0.00). As expected from the protective effect of immunity among the piglets with low HL, individuals with specific antibodies (IM) exhibited a lower probability of becoming viremic compared to individuals without specific antibodies (SU) (T_IM-VIR_ ∼0.00 lower than T_SU-VIR_ = 0.297; 95% CI [0.154; 0.495]). However, among the piglets with medium-high HL, we did not observe any difference in becoming viremic between individuals with (IM) or without (SU) antibodies (T_IM-VIR_ = 0.042; 95% CI [0.010; 0.155] was similar to T_SU-VIR_ = 0.044; 95% CI [0.011; 0.165]). The contrast between susceptible and immune individuals (SU and IM) was thus observed only when individuals had low levels of HL.

## Discussion

Our analysis showed that HA and HL levels are variable both within and among juvenile wild boar individuals. HA also varied according to piglets' age and between populations. HA levels were initially lower, but increased more quickly in PPNR compared to BMC. Piglets having a high relative body condition exhibited high HA and HL levels. During the CSFV outbreak, HA and HL levels were negatively correlated to the probability of becoming viremic. Furthermore, the probabilities of becoming viremic and of acquiring specific antibodies differed between groups of piglets with high and low levels of HL, independently of individuals' age. Consecutively, the highest survival was observed in the piglets with highest average HL levels.

The variations in HAHL titres in 2006 suggest natural variability of Nab concentration and Cp activity among growing piglets. Taken into account individual differences in initial HA values among individuals, partly explained by their body condition, HA increased with piglets' age, such as observed in other vertebrate species [38], [39], [40]. Our results show that Nabs levels were initially lower, but increased faster, in PPNR than in BMC. Since piglets from BMC exhibited the highest growth rate, this result suggests a possible trade-off between the mounting of the immune response and individual growth, such as proposed by Lochmiller and & Deerenberg [1] and demonstrated by Mauck et al. [15] in the Storm petrel (*Oceanodroma leucorhoa*). This result also suggests that wild boar piglets quickly develop the non specific component of their immune function even though resources are limited, such as in PPNR. This rapid development of innate immunity may be a useful strategy [1] for this ubiquitous and short-living ungulate species [41] that is faced with many pathogens [19]. Differences in host genetic background [12] or exposure to antigens could also contribute to different Nabs levels among individuals and areas [40], [42].We cannot therefore exclude the possibility that the HLHA status of the individual animals was influenced by the area in which the CSFV outbreaks occurred, as these two areas experienced CSFV outbreaks during different time periods with possible different intensity.

The correlation between HL and HA was expected since the lysis of exogenous red blood cells by complement is activated by the formation of antigen-Nab complexes, *i.e.*, the agglutination of chicken red blood cells in the present study [1], [14]. After correcting for this effect, HL was correlated to relative body condition, but no longer to age or area. These results suggest that Cp activity increase with age as a result of an increasing concentration of Nabs-antigen complexes, while the concentration of the complement proteins was relatively stable during piglet growth. The correlation between Cp activity and body condition was consistent with previous observations from Gilot-Fromont et al. [5] in roe deer (*Capreolus capreolus*).

We observed a negative correlation between HA and HL levels and the subsequent probability of becoming viremic, suggesting a potential protective effect of Nab and Cp against viremia. Given the gradual increase of Nabs with age and relative body condition, these results also correspond to a higher risk to become viremic in youngest and smallest individuals, which is consistent with the particular susceptibility of young individuals to CSFV [23].

Moreover, CMR modelling indicated that individual difference in Cp activity (conserved along captures and thus independent on age) resulted in different resistance levels to CSFV infection: piglets with high Cp activity were less likely to become viremic and developed specific antibodies more often than piglets with low Cp activity. As a result, survival also differed between the two groups of piglets. These results obtained in a longitudinal framework suggest that Nabs and Cp levels are a cause, or a relevant indicator, of the capacity to recover from infection and develop specific antibodies. Regarding the mechanisms of Nabs and Cp activity, it is unlikely that Nabs and Cp could prevent first infection since CSFV multiply in the tonsil before invading the blood [23]. However an accelerated elimination of CSFV from the blood of infected individuals having high HAHL levels is a possible scenario: the clearing action of Nabs and Cp in the blood was demonstrated for other pathogens in mice and chicken [11], [12]. Furthermore, the Nabs and Cp are known to activate the lymphoid cells and the development of specific immunity, thus individuals with high CHI levels would be expected to develop a strong specific antibody response [1], [11].

Although CHI is a relevant proxy to explain disease outcome, its determinants could be further explored, in particular regarding the maternal or genetic effects. Maternal immunity transmitted by sows to their offspring is known to facilitate piglets' recovery from CSFV infection [20], [21]. However, besides its direct protective effect, maternal immunity influences the ability of offspring to respond through adaptive responses [43]. Here, we observed a lower probability of becoming viremic in individuals with antibodies (IM) compared to individuals without antibodies (SU) among the piglets with low HL. But this protective effect of specific antibodies was no longer significant among the piglets with medium-high HL. This result suggests that the selective benefit of developing a medium-high HL level (Cp activity) was more obvious in the absence of maternally derived antibody (MDA), *i.e.*, in a phase of disease emergence with a low proportion of sows having yet experienced infection and developed specific immunity. The mechanisms of such interaction between CHI and MDA should be clarified. The natural variability of CHI among piglets may also be linked to genetic differences, such as demonstrated in chicken lineages [12], and may contribute to the variable susceptibility to CSFV observed among domestic pig breeds [24]. Further studies are thus required to test the links between genetic background, maternal effects and CHI variations, and the mechanisms for subsequent resistance to pathogens.

## Conclusions

This longitudinal study highlights the strengths of using a simple HAHL assay on serum for studying CHI within wildlife populations. We explored the development of CHI in wild boar piglets and its variations among individuals and areas. Using an intense capture-mark-recapture process, this study demonstrates for the first time a correlation between constitutive humoral immunity within a wildlife population and individual resistance to/recovery from a lethal pathogen. Future studies should be dedicated to explore the links between immunity, genetics and individual resistance to infections within natural populations, since the variation in individual disease outcome may influence host-pathogen dynamics [44], [45]. Different CHI patterns among populations, either driven by genetic or environmental factors, could thus generate spatial differences in disease dynamics and pathogen impact on host fitness [46]. Exploring the genetic drivers of CHI variability could also highlight some of the selective pressures generated by pathogens on their hosts [47], [48].
